# Knockdown of zebrafish YY1a can downregulate the phosphatidylserine (PS) receptor expression, leading to induce the abnormal brain and heart development

**DOI:** 10.1186/s12929-016-0248-1

**Published:** 2016-02-29

**Authors:** Wei-Lun Shiu, Kuan-Rong Huang, Jo-Chi Hung, Jen-Leih Wu, Jiann-Ruey Hong

**Affiliations:** Laboratory of Molecular Virology and Biotechnology, Institute of Biotechnology, National Cheng Kung University, Tainan, 701 Taiwan, ROC; Laboratory of Marine Molecular Biology and Biotechnology, Institute of Cellular and Organismic Biology, Academia Sinica, Nankang, Taipei, 115 Taiwan, ROC

**Keywords:** YY1a, Phosphatidylserine receptor, Zebrafish, Knockdown, Brain, Heart

## Abstract

**Background:**

Yin Yang 1 (YY1) is a ubiquitously expressed GLI-Kruppel zinc finger-containing transcriptional regulator. YY1 plays a fundamental role in normal biologic processes such as embryogenesis, differentiation, and cellular proliferation. YY1 effects on the genes involved in these processes are mediated via initiation, activation, or repression of transcription depending upon the context in which it binds. The role of the multifunctional transcription factor Yin Yang 1 (YY1) in tissue development is poorly understood. In the present, we investigated YY1a role in developing zebrafish on PSR-mediated apoptotic cell engulfment during organic morphogenesis.

**Results:**

YY1a is first expressed 0.5 h post-fertilization (hpf), in the whole embryo 12 hpf, and in brain, eyes, and heart 72 hpf by in situ hybridization assay. The nucleotide sequence of zebrafish YY1a transcription factor (clone *zfYY1a*; HQ 166834) was found to be similar to that of zebrafish YY1a (99 % sequence identity; NM 212617). With the loss-of-function assay, YY1a knockdown by a morpholino oligonucleotide led to downregulation of the phosphatidylserine engulfing receptor zfPSR during embryonic segmentation and to the accumulation of a large number of dead apoptotic cells throughout the entire early embryo, especially in the posterior area. Up to 24 hpf, these cells interfered with embryonic cell migration and cell-cell interactions that normally occur in the brain, heart, eye, and notochord. Finally, with gain-of-function assay, defective morphants could be rescued by injecting both *YY1a* mRNA and *PSR* mRNA and trigger resumption of normal development.

**Conclusions:**

Taken together, our results suggest that YY1a regulates PS receptor expression that linked to function of PSR-phagocyte mediated apoptotic cell engulfment during development, especially the development of organs such as the brain and heart. YY1a/PSR-mediated engulfing system may involve in diseases.

## Background

Yin Yang 1 (YY1; also called delta, NF-E1, and UCRBP) is a ubiquitously expressed GLI-Kruppel zinc finger-containing transcriptional regulator [1–4]. YY1 plays a fundamental role in normal biologic processes such as embryogenesis, differentiation, and cellular proliferation [5]. Its effects on the genes involved in these processes are mediated via initiation, activation, or repression of transcription depending upon the context in which it binds. The activation or repression can be direct via the disruption of binding sites or the causing of changes in DNA conformation or indirect via cofactor recruitment [6–12].

Since its original isolation, YY1 has been shown to regulate an ever-growing number of viral and cellular genes, including the human immunodeficiency virus type 1 and human papillomavirus oncogenes *E6* and *E7*, several proto-oncogenes (*c-myc*, *c-fos*, and *erb2*), cdc-6, the DNA replication-dependent histone H3.2 gene, and others [13–16]. Transient transfection and/or in vitro approaches have been used to characterize YY1 target genes, most of which have yet to be validated as YY1 target genes in vivo. Nevertheless, these studies suggest important roles for YY1 in the control of cell growth, apoptosis, and oncogenic transformation.

Several lines of evidence suggest a critical requirement for YY1 in embryonic development, morphogenesis, and organogenesis. The *Drosophila* counterpart of YY1, *pleiohomeotic (pho)*, participates in embryonic patterning; mutations of pho result in homeotic transformations associated with misexpression of homeotic genes [17–19]. YY1 is also essential for neural induction and patterning in *Xenopus laevis* [20]. Constitutive ablation of YY1 in mice results in peri-implantation lethality [21]. Mice heterozygous for the YY1 gene (yy1^+/−^) display a mild developmental delay and a subset of these animals exhibit neurulation defects and exencephaly [21]. While these observations strongly suggest that YY1 acts during late embryogenesis, early embryonic lethality caused by constitutive loss of function has precluded investigation of the YY1 requirement at later developmental stages [22]. Then, it was shown that YY1 overexpression can reduce fetal troponin (ssTn1) expression in neonatal myocardium [23] and promotes mesodermal cardiac differentiation [24]. Recently, in the zebrafish system, YY1a have examined on promoting the zebrafish liver steatosis and lipotoxicity by inhibiting CHOP-10 exoression [25] and regulating the zebrafish hematopoiesis by beta-arrestin 1 via binding to YY1b and relieving polycomb group repression [26]. Whether the YY1a involve in early embryonic development is still uncover.

Apoptotic cell death occurs by a mechanism that has been evolutionarily conserved from nematodes to humans [27]. In vivo, apoptotic cells are typically engulfed and degraded by phagocytes [28]. Removal of apoptotic cells suppresses inflammation, modulates the macrophage-directed deletion of host cells, and regulates the immune response [29]. The phagocyte engages the dying cells through surface receptors that include the phosphatidylserine receptor (PSR) [30–32], Fc receptor, complement receptors 3 and 4, the ABC1 transporter, members of the scavenger-receptor family [33–36].

Morpholinos are chemically modified oligonucleotides with base-stacking abilities similar to those of natural genetic material [37]. Morpholinos have been shown to bind to and block translation of mRNA in zebrafish cells [38].

Little is known regarding the part played by YY1a in the clearance of cell corpses in zebrafish. In this present, our aim was to define the genetic requirements for a potentially distinct death paradigm involving YY1a in zebrafish during early embryonic development.

## Methods

### Maintenance of fish embryos in culture

Techniques for the care and breeding of zebrafish have been previously described in detail [39]. Embryos were collected from natural matings and maintained in embryo medium (15 mM NaCl, 0.5 mM KCl, 1 mM CaCl_2_, 1 mM MgSO_4_, 0.15 mM, 0.05 mM Na_2_HPO_4_, 0.7 mM NaHCO_3_) at 28.5 °C. Embryos were staged according to standard morphological criteria [40].

### *zfYY1a* cloning

Synthesis and amplification of cDNA was carried out using the SuperScript One-Step™ reverse transcriptase-polymerase chain reaction (RT-PCR) system kit (Invitrogen, Carlsbad, CA) according to the manufacturer’s instructions. YY1 primers P1 and P2 were each added to a final concentration of 0.2 μM. PCR cycling conditions were 54 °C for 30 min, 2 min at 94 °C (to inactivate the reverse transcriptase), 95 °C for 30 s (DNA denaturation), 57 °C for 30 s (annealing), and 72 °C for 60 s (extension) for a total of 35–40 cycles. The RT-PCR primers—zebrafish YY1 P1: 5′-ATGGCGTCGGGCGAGACACTGT-3′ (22mer) and YY1 P2: 5′-TCACTGATTGTTCTTAGCTTTCGCGTGTGT-3′ (30mer)—were used to amplify a fragment covering the complete mRNA region of YY1a. The purity and size of the amplified product were verified by 1.5 % agarose gel electrophoresis and staining with ethidium bromide [41]. The 1074-bp, double-stranded cDNA was purified using the QIAquick™ gel extraction system (Qiagen, Valencia, CA) and subcloned using a pGEMT-easy cloning system (Promega, Madison, WI). The cloned PCR products were sequenced by the dye termination method using an ABI PRISM 477 DNA sequencer (Applied Biosystems, Foster City, CA) and their sequences were scanned against the GenBank database BLAST (www.ncbi.nlm.nih.gov/) and PROSITE (psort.ims.u-tokyo.ac.jp/) programs.

GenBank was the source of the human homologue (accession number NM 003403) and mouse homologue (accession number NM 009537), and the complete sequences for *Xenopus* homologue (accession number NM 001093935), *D. melanogaster* homologue (accession number XM 002099609), and zebrafish homologue (accession numbers NM 212617 and HQ166834).

### Morpholinos

All morpholinos (Gene Tools, LLC, Corvallis, OR) were arbitrarily designed to bind to sequences flanking and including the initiating methionine. We selected sequences based on design parameters recommended by the manufacturer (21–25mer antisense), and looked for the presence of each designed sequence elsewhere in the genome [37, 38]. Sequences were as follows (the sequence complimentary to the predicted start codon is underlined in all cases): YY1-MO-5′-gTACAgTgTCTCgCCCgACgCCAT-3′.

Control-MO-5′-5′-gTACACTgACgCgCgCgTCgCCAT-3′.

(The five sites in the control-MO sequence that are subject to point mutation in the YY1-MO sequence are shown in shaded blocks.)

### Injection of *YY1a* morpholino oligonucleotides

Morpholino oligonucleotides were dissolved in water at a concentration of 1 mM and diluted with water to 0.5, 0.25, and 0.125 mM prior to injection (1.5–3 nl) into the yolk [42].

### In-situ hybridization

Digoxigenin-labeled antisense RNA probes were synthesized from linearized DNA templates containing *YY1a*, *psr*, *pax2a*, and *nkx2.5*, using T7 RNA polymerase (Boehringer Mannheim, Mannheim, Germany). Whole-mount in-situ hybridizations were performed as previously described [43]. In-situ hybridization was used to analyze *YY1a* mRNA expression in embryos injected with YY1-MO or control-MO at 0.5 h post-fertilization (hpf), 12 hpf, 24 hpf, 48 hpf, and three days pf (3 dpf).

### Apoptotic cell staining during embryonic development

Embryos at the one- or two-cell stage were injected with YY1-MO (25 ng) or control-MO (25 ng) or YY1-MO (25 ng) plus PSR mRNA (1 ng), harvested at 18.5 hpf, fixed with 4 % paraformaldehyde in PBS (pH 7.4) at room temperature for 30 min, stained with acridine orange (1 μg.ml^−1^; 3–5 min), washed twice in PBS, and evaluated under a fluorescence microscope (using a 488-nm filter for excitation and a 515-nm long-pass filter for detection) [44]. For the TdT-dUTP labeling step, the embryos were fixed in paraformaldehyde at the end of the incubation period (12 and 24 hpf), dechorionated, incubated in blocking solution (0.1 % H_2_O_2_ in methanol, 30 min, room temperature), rinsed with PBS, incubated on ice in 0.1 % Triton X-100 in 0.1 % sodium citrate for 30 min to increase permeability, rinsed twice with PBS, treated with TUNEL reaction mixture (50 μl; in-situ cell death-detection Kit, Boehringer Mannheim), incubated in a humidified chamber for 60 min at 37 °C, and evaluated for fluorescence-positive apoptotic cells under a fluorescence microscope equipped with a spot II CCD camera (Diagnostic Instruments, Inc., Sterling Heights, MI).

### Western blotting

Embryos were injected with YY1-MO or control-MO at the one- or two-cell stage, harvested 24 hpf, and lysed in 150–200 μl of sodium dodecyl sulfate (SDS) sample buffer (0.63 ml of 1 M Tris–HCl, pH 6.8, 1.0 ml of glycerol, 0.5 ml of ß-mercaptoethanol, 1.75 ml of 20 % SDS, 6.12 ml of H_2_O in 10 ml total). Protein (40 μg) from 24-hpf embryos was loaded onto each lane. Standard western-blot analysis was conducted using human anti-YY1 Mab (Cascade Bioscience, Winchester, MA), anti-zebrafish PSR N-terminus polyAb (self-made), and mouse anti-actin Mab (Chemicon, Temecula, CA). PSR was visualized using horseradish peroxidase-conjugated anti-mouse immunoglobulin (IgG) and the ECL detection kit (Amersham Pharmacia Biotech, Denmark) [44].

### Microinjection of *PSR* mRNA rescues YY1a-mediated defective morphants

Zebrafish *YY1a* and *PSR* [31] were cloned into the pCDNA3 vector, which contains a T7 RNA polymerase promoter site. Linearized plasmid DNA was used as a template for in vitro transcription carried out using the Message Machine Kit (Ambion Inc, Austin, TX), according to the manufacturer’s instructions. For rescue of defective morphants, either 0.1 nl of 200 ng/μl mRNA encoding soluble *YY1a* mRNA or 0.1 nl of 80 and 40 ng/μl mRNA encoding soluble *PSR* mRNA and YY1-MO (0.5 μM) were injected into the one-cell stage of each embryo using a gas-driven microinjector (Medical System Corp, Greenvale, NY) [42].

### Quantitative (q)RT-PCR

Embryos at the one- or two-cell stage were injected with YY1-MO (25 ng) or control-MO (25 ng) or YY1-MO (25 ng) plus PSR mRNA (1 ng), harvested at 18.5 hpf. The mRNA expression levels were measured by a qRT-PCR with a Roche Lightcycler Nano (Roche, Penzberg, Germany). The final volume in a well was 20 ul, and contained 4 ul of OmicsGreen qPCR 5X master mix (Omics Bio, Taipei, Taiwan), 3.2 ng of cDNA, and 50 nM of primer pairs. Cycling parameters were as follows: 95 °C × 900 s, then 45 cycles of the following 95 °C × 15 s, 60 °C × 20 s and 72 °C × 20 s. The standard curve of each gene was checked in the linear range with ß-actin as internal controls. The primer sets are given as follows: P53 primer-forward 5′-ACCACT GGGACCAAACGT AG-3′; Primer-reversed - 5′-CAGAGTCGCTTCTTCCTTCG-3′.

## Results

### *YY1a* cloning and determination of functional domains

**Fig. 1:**
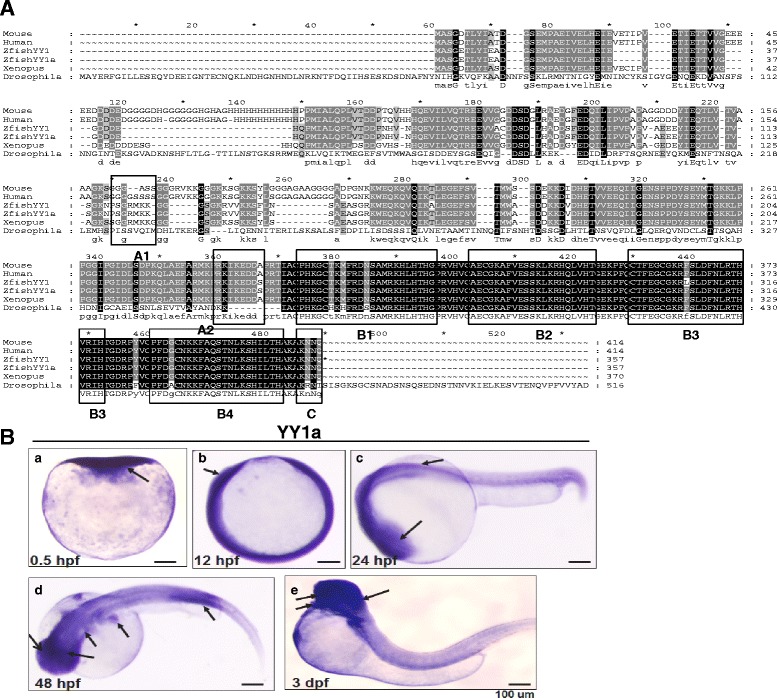
Sequence alignment of zebrafish *YY1a* and its expression profile. **a** A sequence alignment of the YY1a protein (where positions of the functional domains are indicated by a series of boxes labeled A–C). Box A contains potential nuclear localization signals (PSGRMKK [118–124 aa] and PRKIKED [227–233 aa]; Fig. 1a, box A1-2). These lie within the predicted intracellular domain (SMART-TMHMM2 program). Box B1–4 contains the potential zinc finger, CH2H2 type, domains (box B1 [CPHKGCTKMFRDNSAMRKHLHTH, residues 241–263, 23 aa], box B2 [CAECGKAFVESSKLKRHQLVH, residues 270–290, 21 aa], box B3 [CTFEGCGKRFSLDFNLRTHVRIH, residues 298–320, 23 aa], and box B4 [CPFDGCNKKFAQSTNLKSHILTH, residues 328–350, 23 aa]), all of which are well characterized as DNA binding modules. Box C (residues 353–356; AKNN for KKXX domain) contains an ER membrane retention signal based on architectural analysis performed with the SMART-TMHMM2 program. **b** Expression of YY1a from early to late developmental stages detected by in-situ antisense RNA hybridization. YY1a was visualized by blue staining. Lateral view of embryos is shown in panels *a*–*c* and *e*; top view is shown in panel *d*; anterior is on the right side. (*a*) One-cell stage (half-hour). *YY1a* is expressed in all cells examined. *b* At 12 hpf, *YY1a* is expressed throughout the embryo. (*c*) At 24 hpf, *YY1a* is expressed throughout the embryo, but is especially concentrated within the brain region (indicated by an arrow) and in the anterior part of the embryo. (*d*) At 48 hpf, *YY1a* is expressed principally in the posterior notochord (indicated by an arrow) and secondarily in the trunk, brain, kidney, and eyes (indicated by arrows). (*e*) At 3 dpf, the *YY1a* is strongly expressed in some major organs (brain, heart, and eyes; indicated by arrows) and mildly expressed throughout the somite. Scale bars denote 100 μm

*YY1a* was cloned using a specific primer from the 24-hpf total mRNA. The sequences of positive clones were used to search GenBank databases. Some significant matches were made after translating the nucleotide sequences (1074 bp) to amino acid sequences (357 aa) (Fig. 1a). The molecular weight (Fig. 1a) for zfYY1, 39.8 kDa, was predicted from its amino acid sequence.

The nucleotide sequence of zebrafish *YY1a* (*zfYY1*) (HQ 166834) had 99 %, 92 %, 98 %, 98 %, and 98 %, respectively, identity with zebrafish YY1a (NM 212617), and the YY1s for *D. yakuba* (XM 002099609), human (NM 003403), mice (NM 009537), and *Xenopus laevis* (NM 001093935). Here, we named *zfYY1* (HQ 166834) gene is equal to YY1a (NM 212617) gene.

In addition, there were two consensus sequences for the nuclear localization signals within YY1a—a 7-amino-acid peptide (PSGRMKK; at 118–124 aa) and a 7-amino-acid peptide (PRKIKED; at 227–233 aa) (Fig. 1a, box A1-2). Domains of potential zinc finger proteins, CH2H2 type, corresponding to residues 241–263 (box B1; CPHKGCTKMFRDNSAMRKHLHTH, 23 aa), 270–290 (box B2; CAECGKAFVESSKLKRHQLVH, 21 aa), 298–320 (box B3; CTFEGCGKRFSLDFNLRTHVRIH, 23 aa), and 328–350 (box B4; CPFDGCNKKFAQSTNLKSHILTH, 23 aa), which are well-known DNA binding sequences, are shown in box B1–4 (Fig. 1a). The sequence shown in Fig. 1a, box C (residues 353–356; AKNN for KKXX domain) was identified as an endoplasmic reticulum membrane retention signal.

### *YY1a* expression pattern

*YY1a* was expressed from the one-cell developmental stage (30 min) to the 3-day pf (3 dpf) larval stage (Fig. 1b, panels a–e). After somite segmentation, *YY1a* was apparent throughout the embryo (Fig. 1b, panels b–d) and the brain (Fig. 1b, panels b–d). At the 3-dpf larval stage, *YY1a* expression was detected in the brain, heart, and eye (Fig. 1b, panel e).

### *YY1a* knockdown affects apoptotic cell accumulation and delays epiboly

**Fig. 2:**
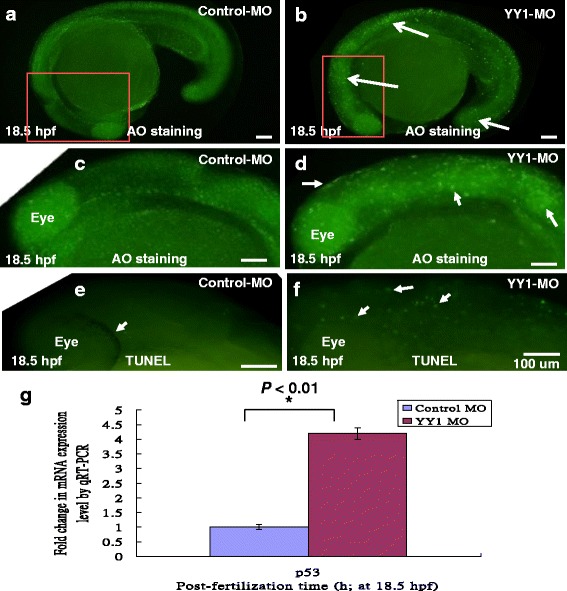
Apoptotic cell analysis by AO staining and TUNEL assay. Control-MO and YY1-MO (25 ng per embryo) were injected at the one-cell stage to block translation of *YY1a* mRNA. The embryos were fixed and observed at the different stages postfertilization (pf). The AO-stained embryos are shown in **a** (Control-MO; 18.5 hpf), **b** (YY1-MO; 18.5 hpf), **c** (Control-MO; 18.5 hpf; enlarged from A), and **d** (YY1-MO; 18.5 hpf; enlarged from B; strongly AO-positive cells indicated by arrows). TUNEL stained embryos (all at 18.5 hpf) are shown in **e** (Control-MO) and **f** (YY1-MO group). Identification of apoptotic cell death-related gene P53 at 18.5 hpf by qRT-PCR approach as a control is shown in (**g**). All data were analyzed using either paired or unpaired Student’s t-tests as appropriate. *P < 0.01. The TUNEL-positive cells under the fluorescence microscope are considered apoptotic. Bars indicate 100 μm

YY1a morpholino oligonucleotides (MO; 25 ng) were injected into embryos to knock down *YY1a* expression (Fig. 2) that morpholino oligonucleotides is localized from the ATG start site for covering the 24 nt. Acridine orange staining revealed the accumulation of corpse cells 18.5 hpf in the brain, interior somite, and tail bar (indicated by arrows, Fig. 2b and d; Fig. 2a and c, the control), while TUNEL staining revealed apoptotic cells throughout the embryo at 18.5 hpf (Fig. 2f; Fig. 2e, the control). Then, we checked the cell death-related gene, P53 by qRT-PCR approach as a control at 18.5 hpf (Fig. 2g) that P53 is also increased 4.2-fold than control group (Control MO).

**Fig. 3:**
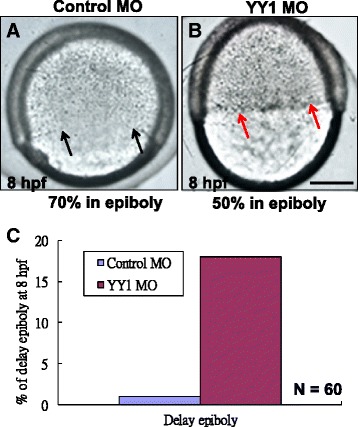
Morpholino-induced knockdown of *YY1a* prevents cell corpse engulfment and cell migration. **a** Control-MO-injected embryos, lateral view of a Control-MO-injected embryo about entering into 70 % epiboly stage at 8 hpf (indicated by arrows) reveals the normally developed. **b** Lateral view of a YY1-MO-injected embryo reveals the delay epiboly about 20 % (indicated by red arrows). Bars indicate 100 μm. **c** Quantification of the delay epiboly embryos from control-MO and YY1-MO injection groups is shown the delay epiboly migration (*N* = 60)

*YY1a* knockdown early in the epiboly stage strongly affected embryonic development at 8 hpf, caused a severe delay in epiboly migration (indicated by red arrows) about 20 % (Fig. 3b; cf Fig. 3a [control MO-injected embryo]), which led to accumulation of some cells in margin layer. Then, the delay epiboly migration embryos was counted that is shown 18 % different (Fig. 3c) in injection embryos (*N* = 60).

### Influence of cell corpses on normal embryonic development

**Fig. 4:**
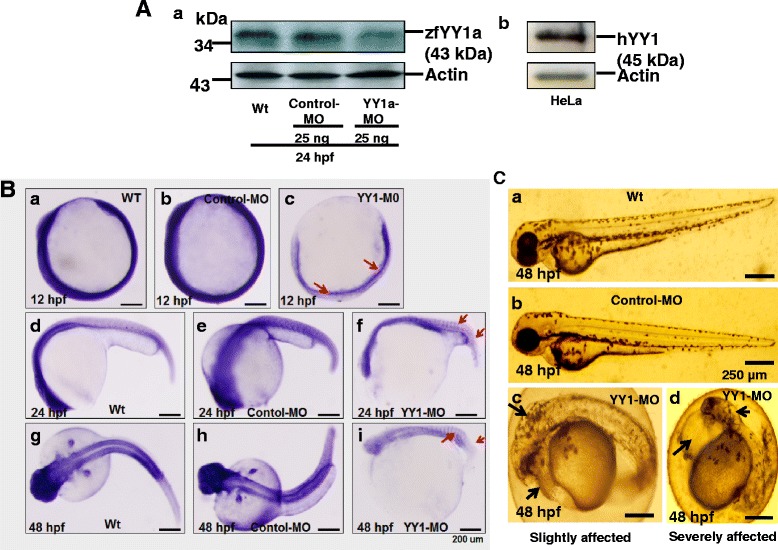
Ubiquitous *YY1a* inhibition by injection of morpholinos (YY1-MO). **a** Western-blot analysis of YY1a proteins in the 24-h stage wild type embryos and YY1-MO-injected embryos. The knockdown of *YY1a* gene in these embryos by YY1-MO injection was shown in panel *a*, lane 3 (panel *a*, lanes 1–3; panel *b*, the positive control, HeLa cell lysate). Actin was the loading control. **b** Each one-cell stage embryo was injected with either 25 ng of control-MO or 25 ng of YY1-MO. At 12, 24, and 48 hpf, embryos were fixed and examined after in-situ hybridization. The phase-contrast images of the wild type (panels, *a*, *d*, and *g*) and control-MO-injected (panels *b*, *e*, and *h*) embryos show normal development and *YY1a* expression patterns. However those of the YY1-MO-injected embryos show abnormal development (panels *c*, *f* and *i*) and delayed *YY1a* expression (panel *c*, 12 hpf; arrows) and abnormal *YY1a* expression (panel *f* and *i*; 24 and 48 hpf, respectively; arrows). **c** Phase-contrast images of wild type embryos (panel *a*), control-MO-injected embryos (panel *b*), YY1-MO-injected embryos that are slightly affected (panel *c*), and YY1-MO injected embryos that are severely affected (panel *c*). The abnormal brain and heart are indicated by arrows. Bars indicate 250 μm

We next tested the ability of the YY1-MO and control-MO to block translation of zfYY1a. As shown in Fig. 4a (panel a), injections of 25 ng of YY1-MO blocked 65 % (lane 3) of the control-MO protein expression (lane 2) and wild-type protein expression (lane 1). The molecular weight of zebrafish YY1a (evident in lanes 1–4) was 43 kDa, which was smaller than that of the positive control (about 45 kDa for HeLa cell lysate; Fig. 4a, panel b). At 12 hpf, the YY1-MO-injected (25 ng) embryos were defective reflecting a delay in epiboly (Fig. 4b, *c*, as indicated by arrows; cf. with Fig. 4b, *a* [wild type] and Fig. 4b, *b* [control MO injection]), a loss of normal morphogenetic capability, and abnormal *YY1a* expression (Fig. 4b, panel f, as indicated by arrows; cf. with Fig. 4b, *d* [wild type] and Fig. 4b, *e* [control MO injection]). These changes were also evident at 48 hpf (Fig. 4b, *i*, as indicated by arrows; cf. with Fig. 4b, *g* and *h* [control]).

The abnormalities at 48 hpf were both mild (just shown mild defects in brain and heart but can hatching out) and severe (severe defects in brain and heart, and did not hatching out) (Fig. 4c, *c* and *d*; cf. with Fig. 4c, *a* and *b* [wild type and control MO-injected]). The mild defects included abnormal mid- and hindbrain morphology or a slight delay in heart development prior to hatching (Fig. 4c, panel c; as indicated by arrows). The severe defects included marked delays in development manifesting as shrinkage of the brain, defects in heart development, and failure to hatch (Fig. 4c, panel d; as indicated by arrows).

YY1-MO dosage from 12.5 ng (*n* = 174), 25 ng (*n* = 178), to 50 ng (*n* = 174) was significantly correlated with developmental change (compare with control-MO injection [*n* = 126]) at 48 hpf as shown in Table 1.

**Table 1:** Percentage of morphant phenotypes during knockdown of YY1a by YY1-morpholines

Phenotype^a^	Control-MO	YY1-MO		
	50 ng	12.5 ng	25 ng	50 ng
Number of embryos	126	174	178	174
Normal type	92.0 %	40.0 %	5.6 %	1.3 %
Weakly defective	8.0 %	41.7 %	10.2 %	3.6 %
Severely defective		8.0 %	20.2 %	7.3 %
Death type		10.3 %	64.0 %	87.8 %

^a^All embryos were examined at 2 dpf

### YY1a knockdown downregulates PSR expression

**Fig. 5:**
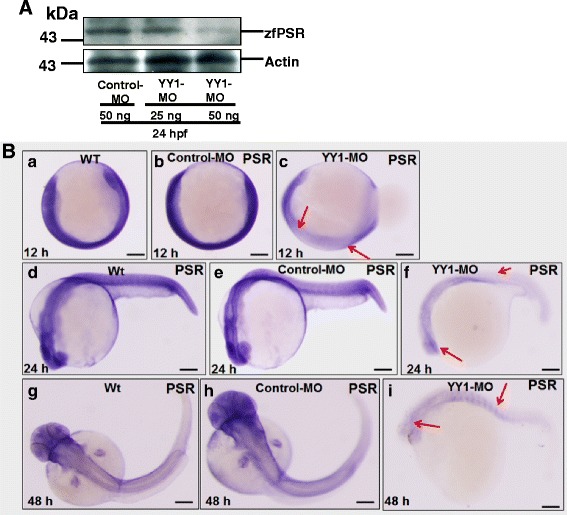
Morpholino-induced knockdown of *YY1a* results in down-regulation of PS receptor at 24 hpf. **a** Western-blot analysis of PSR proteins in lysates of YY1-MO-injected embryos. Note that PSR protein level in these embryos decreases in a YY1-MO dose-dependent manner (Fig. 5a, panel *a*, lane 1 [control-MO, 50 ng]; lane 2 [YY1-MO, 25 ng], and lane 3 [YY1-MO, 50 ng]). Panel *b* shows the actin loading control. **b** One-cell stage embryos were each injected with either 25 ng of control-MO or 25 ng of YY1-MO. At 12, 24, and 48 hpf, embryos were fixed and in-situ hybridization was performed as described in the Methods section. Phase-contrast images show normal development and *psr* expression patterns in wild type (panels *a*, *d*, and *g*) and control-MO-injected (panels *b*, *e*, and *h*) embryos but abnormal development (panels *c*, *f*, and *i*) and either a mild (panel *c*, 12 hpf) or severe (panel f, 24 hpf; panel i, 48 hpf) delay in *psr* expression (arrows) in YY1-MO-injected embryos. Bars indicate 100 μm

To determine whether YY1a knockdown affects expression of PSR (a mediator of apoptotic cell engulfment by macrophages) was investigated. As shown in Fig. 5a, injections of 25 and 50 ng of YY1-MO blocked 50 % (lane 2) and 90 % (lane 3) of PSR expression (compared with lane 1 [control MO, 50 ng, normal control]) at 24 hpf.

We found that YY1-MO (25 ng) injection apparently reduced PSR expression throughout the embryo (Fig. 5b, *c*, as indicated by arrows; cf. with Fig. 5b, *a* [wild type] and Fig. 5B, b [control MO injection]) at 12 hpf and reduced PSR expression in brain and somites (Fig. 5b, *f*, as indicated by arrows; cf. with Fig. 5b, *d* [wild type] and Fig. 5b, *e* [control MO injection]) at 24 hpf. The changes at 24 hpf were still present at 48 hpf (Fig. 5b, *i*, as indicated by arrows; cf. with Fig. 5b, *g* and *h* [control]).

### *YY1a* involvement in brain and heart organogenesis

**Fig. 6:**
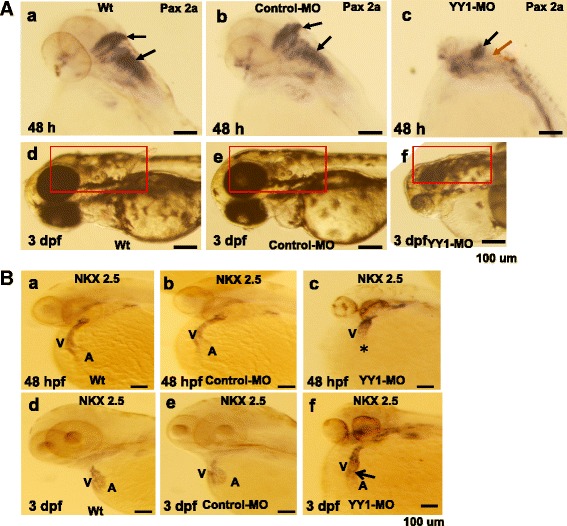
Morpholino-induced knockdown of *YY1a* results in brain and heart defects. Morphological analysis of embryos injected with either 25 ng of control-MO or YY1-MO and examined at 48 hpf or 3 dpf following staining with *pax 2a*, *nkx 2.5*, or no stain. **a** Panels *a*–*c*. The embryos are stained with *pax 2a* (*a*, *b*; top view, anterior to the right) or not stained (*d*–*f*; lateral view). The YY1-MO-injected embryos (panel *c*) has smaller brain (indicated by black arrow) and abnormal *pax 2a* pattern (indicated by red arrow) as compared with wild type embryos (panel *a*) and control-MO-injected embryos (panel *b*). The fore-, mid-, and hindbrains are shorter (indicated by open square [panel *f*]; cf. with control [panels *d* and *e*]). **b** Staining with *nkx 2.5* was used to monitor heart development. Normal heart formation was delayed at 48 hpf (panel *c*; indicated by star). Compare with the atria (A) and ventricles (V) in panels *a* and *b*. The tube-like heart in panel *f* (indicated by arrows) should be compared with that in wild type and control-MO-injected embryos (panels *d* and *e*, respectively) at 3 dpf. Bars indicate 100 μm

Brain and heart development was monitored using marker genes for the brain *pax 2a* [45] and heart *nkx 2.5* [46] at 24, 48, and 72 hpf. Significant differences were apparent at 12 and 24 hpf (data not shown). In brain development study, the developing mid- and hindbrains appeared to shrink and express abnormal amounts of *pax 2a* at 48 hpf (Fig. 6a, *c*; as indicated by arrows; cf. with Fig. 6a, *a* [wild type] and Fig. 6a, *b* [normal control]). At 3 dpf, YY1-MO morphants were apparently shorter and their mid- and hindbrains apparently smaller by about 2-fold (Fig. 6a, *f*; cf. with Fig. 6a, *d* [wild type] and Fig. 6a, *e* [control MO injection]).

In heart development study, probing with *nkx 2.5* allowed the monitoring of heart morphogenesis. In-situ assay using *nkx 2.5* as probe detected a severe delay in heart development (atria absent from a tube-like heart; Fig. 6b, *c*; cf with Fig. 6b, *a* [wild type] and *b* [control MO injection]) at 48 hpf and presence of a tube-like heart (twice the normal size in length) and abnormal blood circulation rate (Fig. 6b, *f*; cf. with Fig. 6b, *d* [wild type] and *e* [control MO injection]) at 3 dpf.

### *YY1a* mRNA rescues defective morphants

The results of 2.5 ng of *YY1a* mRNA prevented the developmental block due to coinjection of YY1-MO with 25 ng of YY1-MO at the embryonic one- or two-cell stage. In the present study, the same result was obtained by injecting 25 ng of YY1-MO and 2.5 ng of *PSR* mRNA.

**Fig. 7:**
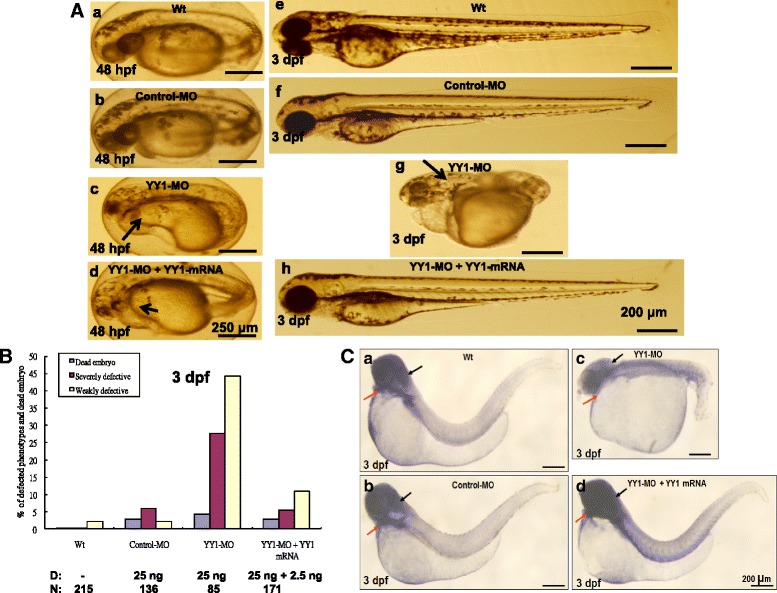
Injection of *YY1a* mRNA rescued embryos from YY1a morpholino-induced defects. YY1-MO (25 ng) and *YY1a* mRNA (2.5 ng) were co-injected at the one-to-two cell stage, and embryos were assessed at 48 hpf and 3 dpf. **a** Panel c shows YY1-MO-injected embryos with severe morphological deformities (indicated by long arrow). After rescue, the deformities were markedly reduced (panel *d*: indicated by short arrow; cf. wild type [panel *a*] and control-MO-injected [panel *b*] embryos). Bars indicate 250 μm. Whole embryos before (panel *g*) and after (panel *h*) rescue can be compared with wild-type (panel *e*) and control-MO-injected (panel *f*) embryos. Bars indicate 200 μm. **b** The ability of *YY1a* mRNA injection to rescue embryos from morphological deformities during development at 3 dpf was estimated. **c** Rescued embryos show normal morphology and normal *YY1a* expression in the brain, heart, and somites and are all stained with the *YY1a* probe at 3 dpf developmental stage. The normal morphology and normal *YY1a* pattern in the brain and heart (indicated by arrows) seen in rescued embryos (panel *d*) is in contrast to the deformed brains and hearts seen in YY1-MO knockdown embryos (panel *c*; indicated by arrows). The wild type (panel *a*) and control-MO-injected (panel *b*) are the negative controls. Bars indicate 200 μm

YY1-MO-injected embryos had mild and severe developmental defects (Fig. 7a, *c*) at 48 hpf. After rescue, embryos had only mild defects at 48 hpf (Fig. 7a, *d*; cf. with Fig. 7a, *a* [wild type] and b [control MO injection]). Both mild and severe defects were evident at 3 dpf Figs. 4c, *c* and *d*) and no defects were evident in the controls (Fig. 7a, *e* [wild type] and *f* [control MO injection]).

Survival and morphant phenotype rates were estimated in wild type (*n* = 215), control-MO (*n* = 136), YY1-MO plus *YY1a* mRNA (*n* = 171), and YY1-MO (*n* = 185) embryos at 3 dpf. The rate of severe defect occurrence was reduced from 27.5 % to 5 % and the rate of mild defect occurrence was reduced from 45 % to 11 % by injection of the *YY1a* mRNA (Fig. 7b). Rescue restored morphogenesis and brain-heart development to normal (Figs. 7c, *d*; cf. with wild type [Fig. 7c, *a*] and control-MO injected embryos [Fig. 7c, *b*]) at 3 dpf. The YY1-MO-injected embryos with slight reduction in brain size and heart development had patterns of morphogenesis and *YY1a* expression that reflected these mild deformities (Figs. 7c, *c*; as indicated by arrows; cf. with Fig. 7c, *a* [wild type] and Fig. 7c, *b* [control MO injection]).

### *PSR* mRNA rescues YY1a-mediated defective morphants

To determine extra reloading PSR mRNA whether can rescue YY1a defected. The 0.5 ng or 1 ng of *PSR* mRNA were used to coinjection of YY1-MO with 25 ng of PSR-MO at the embryonic one- or two-cell stage.

**Fig. 8:**
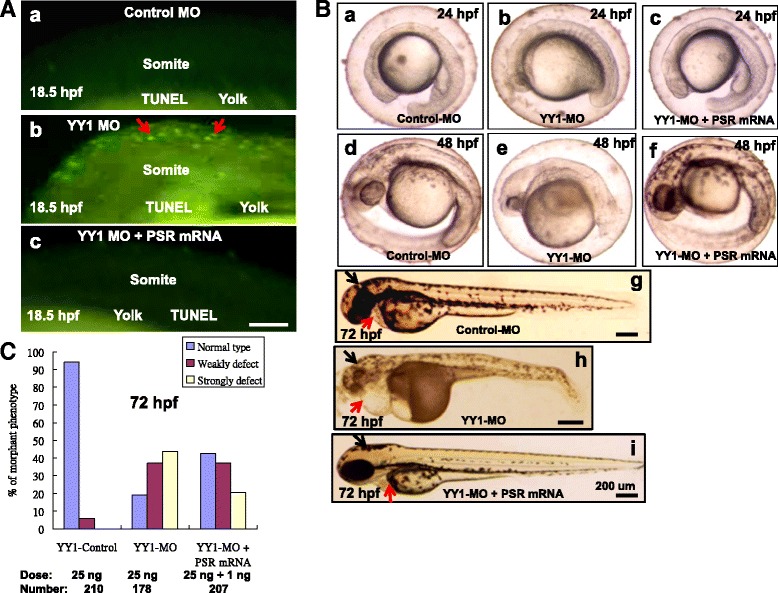
Injection of *PSR* mRNA rescued embryos from YY1a morpholino-induced defects. YY1-MO (25 ng) and *PSR* mRNA (1 ng) were co-injected at the one-to-two cell stage, and embryos were assessed at 18.5 hpf, 48 hpf and 3 dpf. **a** Apoptotic cell analysis by TUNEL assay. Control-MO and YY1-MO (25 ng per embryo) or YY1-MO (25 ng per embryo) plus *PSR* mRNA (1 ng) were injected at the one-cell stage to express extra PS receptor. The embryos were fixed and observed at the different stages postfertilization (pf). TUNEL stained embryos (all at 18.5 hpf) are shown in A:a (Control-MO), A:b (YY1-MO group) and A:c (extra PSR mRNA group). The TUNEL-positive cells under the fluorescence microscope are considered apoptotic, especially in A:b (indicated by arrows). Bars indicate 100 μm. (B) Rescued embryos show normal morphology in the brain, heart, and somites. The normal morphology in the brain and heart (indicated by arrows) seen in rescued embryos (panel *c*, at 24 hpf; *f*, at 48 hpf; *i*, at 72 hpf) is in contrast to the deformed brains (indicated by black arrows) and hearts (indicated by red arrows) seen in YY1-MO knockdown embryos (panel *b*, at 24 hpf; *e*, at 48 hpf; *h*, at 72 hpf; indicated by arrows). The control-MO-injected (panel *a*, at 24 hpf; *d*, at 48 hpf; *g*, at 72 hpf) are the negative controls. Bars indicate 200 μm. **c** The ability of *PSR* mRNA injection to rescue embryos from morphological deformities during development at 72 hpf was estimated

The rescued group (1 ng of PSR mRNA is better than 0.5 ng) was co-injected into embryos with 25 ng of YY1-MO (Fig. 8a). TUNEL staining revealed the accumulation of corpse cells 18.5 hpf in the whole embryo, especially the somite (Fig. 8a:*b*; indicated by arrows) in YY1-MO group, when compared with Wild type (Fig. 8a:*a*) and *PSR* mRNA-rescued group (Fig. 8a:*c*), which also shown the very few accumulation of corpse cells in the somite (indicated by arrow).

YY1-MO-injected embryos had severe developmental defects (Fig. 8b:*b*, *e* and *h*) at 24 h, 48 h and 72 hpf, respectively. After rescue, embryos had just mild defects at 72 hpf in the heart (Fig. 8b:*i*; indicated by arrow). And no defects were evident in the controls (Fig. 8b, *g* [control MO injection]).

After rescued by PSR mRNA, the normal type (Fig. 8c; 3 dpf) and morphant phenotype rates were estimated in control MO (*n* = 210), YY1-MO (*n* = 178), and YY1-MO plus PSR mRNA (1 ng; *n* = 207) embryos at 3 dpf. The rate of normal type occurrence was significantly increased from 19 % to 42 % (1 ng of mPSR group), which can enhance up to 23 % in normal type. The rate of mild defect occurrence was the same from 38 % to 38 % and the rate of severe (strongly) defect occurrence was reduced from 43 % to 20 % by injection of the *PSR* mRNA (Fig. 8c), which can reduce up to 23 % that was shown up (YY1a)-and-downstream (PSR) relationship.

## Discussion and conclusion

Phagocytosis plays an important role in inflammation and autoimmune responses [29], [47, 48], and in remodeling of tissue during development [49]. The link between phagocytosis and development thus provides a useful system to identify genes involved in phagocytosis. These genes have been difficult to identify in mammalian systems [50]. While some questions remain, PSR appears to play a central role in the clearance of apoptotic cells [51]. In the present, transcriptional factor YY1a played a positively regulator in the PSR gene expression that PSR-mediated engulfment of cell corpses required for normal embryonic development and tissue morphogenesis.

### PSR and others receptors involved in cell corpse engulfment during development

The phagocytic removal of apoptotic cells suppresses inflammation, modulates the macrophage-mediated deletion of host cells, and critically regulates the immune response [29] in higher organisms. In vertebrates, the phagocyte engages dying cells through specific receptors such as the phospatidylserine receptor (PSR) [30–32]—a professional receptor in zebrafish, but not in Drosophila system. Some receptors involved in phagocytosis (e.g., Fc receptors, complement receptors 3 and 4, the ABC1 transporter, members of the scavenger-receptor family [33], and BAI1 [34] are not expressed in the early embryonic stage. Recently, the gene encoding *Satblin-2* receptor [35] was found to be expressed very early in embryonic development (before 5.4 hpf), but whether regulation by YY1 influences its expression remains unknown. On the other hand, a new corpse engulfment receptor TIM4 [37] was found to be expressed late (72 hpf) but not early in the embryonic development of zebrafish, so this receptor may not be involved in early corpse engulfment. In our system, we found that PSR-mediated death cell clearing is important for normal development and especially on brain and heart development that can regulate by YY1a in the up-streaming.

### YY1a knockdown can affect dead cell engulfment and embryonic phenotype

In our present, first reported abnormal development after knockdown of *YY1* was monitored over time. Three embryonic phenotypes (mildly defective, severely defective, and dead embryos) could be differentiated at 12, 24, 48, and 72 hpf. Mild defects (enlarged heart cavity and brain malformation) were apparent at 48 hpf but not at 12 hpf. Severe defects at 12 hpf were associated with accumulation of a large number of apoptotic corpses throughout the embryo that interfered with epiboly and posterior development and their persistence at 48 hpf led to failure to hatch and death at 3 dpf. This pattern resembled that of embryos subjected to PSR knock down [31]. Interestingly embryos could be prevented from developing mild and severe defects by injecting YY1 mRNA at 48 h and 72 hpf (Fig. 7) and PSR mRNA at 24 h, 48 h and 72 hpf (Fig. 8b and c) as a downstream regulating gene, indicating that such an injection could potentially be used to correct *YY1a* gene defects.

### New role of YY1a affect PSR expression on engulfing apoptotic cell function

Recently, it was shown that YY1 can direct targeting by miR-34a [52]. Constitutive ablation of YY1 in mice has been reported to result in peri-implantation lethality [21]. Heterozygous mice (yy1^+/−^) display a mild delay in development, and a subset of these animals exhibit neurulation defects and exencephaly [21]. While these observations strongly suggest that YY1 acts during late embryogenesis, the early embryonic lethality caused by constitutive loss of function precludes investigation of the YY1 requirement at later developmental stages [22]. Furthermore, DNA array analysis of the response to conditional allele knockout of YY1 gene in mouse embryonic fibroblasts [22] identified about 135 up- and downregulated genes involved in (1) cell cycle control, (2) mitosis and cytokinesis, (3) DNA replication and repair, (4) apoptosis, (5) cell growth and proliferation, and (6) development. In zebrafish system, we found that zfYY1a knockdown can reduce PSR mRNA and protein expression levels. Furthermore, we found that zfYY1a-mediated defected could be rescued by mPSR injection at 24 h, 48 h and 72 hpf (Fig. 8b and c). Thus, YY1 may play a new role that act as a positive regulator of PSR in the up-streaming during early embryonic development.

### Why is important of YY1a-mediated PSR clearing system on brain and heart morphogenesis

The normal development is important for tissues or organs completely formation that involved in turn on the programmed cell death and engulfing of corpses cell system, those events were tightly control and regulated, if somewhere connection was mistaken or failed that also caused the abnormal development and diseases. We proposed that YY1a/PSR-mediated engulfing system is important for this role.

The above-mentioned evidence supports our idea that the accumulation of cell corpses interferes with normal embryonic development by altering cell movement (Figs. 3b and c) in early epiboly stage during normal genetic program process.

From the developmental view, at the mid-blastula stage (5.25 hpf), the gastrula starts to emerge, all cells acquire the ability to move [40] (ZFIN website), and programmed cell death/apoptosis is turned on [31]. Development requires movement of cells that several types of movement occur including epiboly, involution, convergence, and extension [53–55]. If the apoptotic cell corpses are not quickly removed by professional engulfing PSR-mediated clearance system [31], they can impede cell movement and delay epiboly and thereby the onset of organ development such as on three germ-layers development for ectoderm, mesoderm and endoderm formations. Cells involved in the development of somites, primordial pharyngea, neuromeres, primary organs, and the tailbud are typically associated with one of three germ layers (ectoderm, mesoderm, or endoderm) [40, 53]. Cell corpses typically affect morphogenesis of normal organs at the pharyngula (24 h), hatching (48 h), and early larval (72 h) stages.

Recently, this is new emergence area that mechanic forces induced by accumulated dead cells [56–58] that the phagocyte engages the dying cells through phosphatidylserine receptors (PSR) and other receptors [30–32]. Here, we found that PSRs are professional receptors for phagocytosis in zebrafish [31] between at 5.4hpf and 6 hpf that PSR-mediated engulfing pathway regulated by YY1a.

### Summary (Fig. 9)

**Fig. 9:**
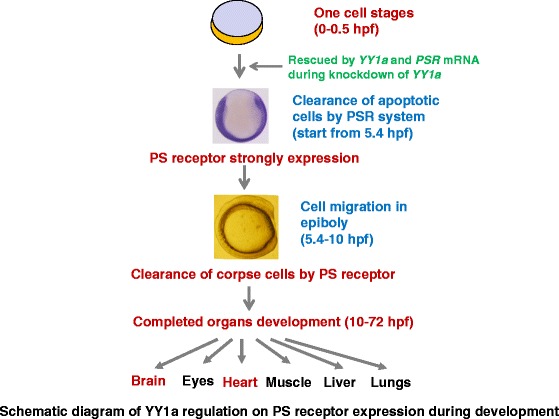
The schematic of our hypothesis on YY1a-mediated PSR engulfing dead cell system affects normal embryonic development and organogenesis. Development begins at the completion of the first zygotic cell cycle (0–0.5 h; YY1a and PSR genes also were expressed), proceeds to the cleavage stage (0.75 h) and then to cell cycles 2–7, which occur rapidly and synchronously. During the gastrula stage, three germ layers are formed (5.25 hpf). At this point, the cell mobility is needed for epiblast, hypoblast, and embryonic axis formation through to the end of epiboly. Apoptosis can occur during the early shield-stage gastrula, but cell corpses are removed promptly through PS receptor-mediated engulfment about starting at 5.4 hpf, which may be regulated by transcription factor zfYY1a. If not removed, the cell corpses accumulate gradually and progressively impede cell movement necessary for triggering downstream developmental events. Cells in the three germ layers (ectoderm, mesoderm, and endoderm) participate in segmentation (10 h), leading to development of somites, pharyngeal structures, primordia, and neuromeres, which is necessary for primary organogenesis and proper appearance of the tailbud. A disturbance at this early stage influences organogenesis at later stages (i.e., the pharyngula [24 h], hatching [48 h], and early larval [72 h] stages). *YY1a* knockdown induced morphant defects that are also corrected by extra *YY1a* mRNA and PSR mRNA injection at one cell or two cell stage

The first traced the transcription factor YY1a as a material factor was expressed from 0.5 hpf to 3 dpf. This factor can regulate downstream molecule PS receptor expression for engulfing of corpse cells during priming the programmed cell death at about 6 hpf. With PSR-mediated engulfing system for completed to clean the death cells during early development that is important for three germ layers formation and then tissue and organs development. If it didn’t smooth process that defected embryos especially on brain and heart developments also found at late development stage. So YY1a/PSR-mediated engulfing system is very important for normal development and may cause diseases.
